# Access to best‐evidenced mental health support for care‐experienced young people: Learnings from the implementation of cognitive therapy for PTSD

**DOI:** 10.1111/bjc.12471

**Published:** 2024-07-16

**Authors:** Rosie McGuire, Richard Meiser‐Stedman, Patrick Smith, Davin Schmidt, Gretchen Bjornstad, Robyn Bosworth, Timothy Clarke, Joe Coombes, Emma Geijer Simpson, Kristian Hudson, Paula Oliveira, John Macleod, Ruth McGovern, Paul Stallard, Katie Wood, Rachel M. Hiller

**Affiliations:** ^1^ Division of Psychology & Language Sciences UCL London UK; ^2^ Department of Clinical Psychology and Psychological Therapies University of East Anglia Norwich UK; ^3^ Institute of Psychiatry, Psychology and Neuroscience King's College London London UK; ^4^ University of Exeter Medical School, University of Exeter Exeter UK; ^5^ Norfolk & Suffolk NHS Trust Norwich UK; ^6^ Population Health Sciences Institute University of Newcastle Newcastle UK; ^7^ Improvement Academy, NIHR ARC Yorkshire & Humber Bradford Teaching Hospitals NHS Trust Bradford UK; ^8^ Anna Freud National Centre for Children & Families London UK; ^9^ NIHR School for Primary Care University of Bristol Bristol UK; ^10^ Department of Health University of Bath Bath UK

**Keywords:** care‐experienced young people, child welfare, foster care, PTSD, CBT, cognitive therapy for PTSD, trauma focused CBT, implementation

## Abstract

**Objectives:**

Rates of PTSD are up to 12 times higher in care‐experienced young people (CEYP) compared to their peers. Trauma‐focused CBTs (tf‐CBT) are the best‐evidenced treatment for youth with PTSD, yet, in practice, CEYP often struggle to access this treatment. We worked alongside services to understand barriers and facilitators of the implementation of cognitive therapy for PTSD (a type of tf‐CBT) to CEYP.

**Design:**

This was an active, open implementation trial.

**Methods:**

We recruited 28 mental health teams across England, including general CAMHS, targeted CAMHS for CEYP and social care‐based teams. From these teams, participants were 243 mental health professionals, from a wide variety of professional backgrounds. Following recruitment/intervention training, teams participated in rolling three monthly focus groups and individual interviews, to understand what helped and hindered implementation. Data were analysed using a framework analysis conducted using CFIR 2.0.

**Results:**

Almost half of the teams were able to implement, but only approximately one quarter with CEYP, specifically. Universal barriers that were discussed by almost all teams particularly highlighted service structures and poor resourcing as major barriers to delivery to CEYP, as well as the complexities of the young person and their network. Unique factors that differentiated teams who did and did not implement included commissioning practices, the culture of the team, leadership engagement and style, and the development of supervision structures.

**Conclusions:**

Findings offer key considerations for mental health teams, service leads, commissioners and policy‐makers to enhance delivery of best‐evidenced mental health treatments like CT‐PTSD, for CEYP.

**Practitioner points**

- Commissioners and service leadership must come together across social care and mental health sectors, to ensure pathways between sectors and services allow CEYP to access needs‐matched best‐evidenced individual psychotherapies.
- When social care and mental health professionals come together to understand the needs and treatment plan for a young person, treatment delivery can be more successful.
- Leadership that supports open and non‐judgmental communication, where anxieties or worries about trauma treatments can be voiced and who prioritizes supervision time is important for teams to implement.
- Teams that buy‐in to evidence‐based practice and CBT approaches, and are motivated to find solutions for delivering the treatment in the face of complexity, are more likely to implement.

## BACKGROUND

Care‐experienced young people (CEYP) often have histories of exposure to interpersonal trauma and significant adversity (Greeson et al., 2011; Hiller, Meiser‐Stedman, et al., 2021). Removal from a biological parent into state care (in the United Kingdom, referred to as local authority care) is most commonly due to abuse or neglect (Department for Education, 2021). There is well‐documented evidence of the high rates of mental health difficulties experienced by CEYP. Around half of children in the care system meet criteria for a diagnosable mental health condition, and complex comorbidities and risks can be common (Bronsard et al., 2016; Ford et al., 2007). Similarly elevated mental health difficulties have also been found in young people who were adopted from care (Minnis et al., 2006). Yet, qualitative work continues to show that CEYP often struggle to access mental health support that meets their needs, both from their own view and that of their caregivers (Hiller et al., 2020; Hiller, Halligan, et al., 2021; Jee et al., 2014; York & Jones, 2017). The unaddressed mental health needs of CEYP are considered one of the key drivers of a range of poor outcomes, including high rates of unemployment, homelessness and ongoing mental health difficulties in adulthood (Jones et al., 2011). There is an urgent need to understand how mental health services can be supported to provide best‐evidenced mental health interventions to CEYP.

Many CEYP have experienced significant trauma before entering care and/or when in the care system (Briggs et al., 2012; Dorsey et al., 2012). Posttraumatic stress disorder (PTSD) is a trauma‐specific mental health condition with rates substantially higher in CEYP than in their peers (Ford et al., 2007), with some estimates suggesting that 30%–55% of young people in care might meet criteria for PTSD (Grasso et al., 2009; Hiller, Halligan, et al., 2021; Hiller, Meiser‐Stedman, et al., 2021; Morris et al., 2015). Without support, PTSD can become chronic and entrenched, and in CEYP, it has been linked to a range of complex comorbidities, aggression and substance misuse (Auslander et al., 2016; Goldstein et al., 2011; Vaughn et al., 2007). While PTSD can be a very difficult condition to live with, there are well‐established interventions that can support a young person to understand and overcome their symptoms. The National Institute for Health and Care Excellence (NICE) have long‐recommended trauma‐focused cognitive behavioural therapy (tf‐CBT) as the first‐line treatment for PTSD, including for young people with maltreatment‐related PTSD (Mavranezouli et al., 2020; NICE, 2018). A recent meta‐analytic review showed that the intervention is as effective for child and adolescent PTSD from complex or multiple traumas, as it is for those exposed the acute or one‐off trauma (Hoppen et al., 2023). Trauma‐focused CBT also remains the best‐evidenced intervention for young people with complex PTSD (Jensen et al., 2022; Sachser et al., 2017).

Despite epidemiological evidence of high rates of trauma and PTSD in CEYP, there is growing evidence that in‐practice PTSD is under‐detected among these young people, with consequences for access to best‐evidenced treatments (Grasso et al., 2009; McGuire et al., 2022). This may partly be driven by not only CEYP under‐reporting their symptoms (Tarren‐Sweeney, 2019) but also potential diagnostic and treatment biases among professionals (McGuire et al., 2022; Woolgar & Scott, 2014). When presented with identical vignettes of a young person with possible PTSD, mental health professionals randomized to a vignette of a young person in care were far less likely to identify PTSD or choose a NICE‐recommended treatment (tf‐CBT or eye movement desensitization and reprocessing [EMDR] therapy), compared to those randomized to the vignette of the young person living with their mother. That is, despite identical symptom descriptions, just identifying a young person as being in care seemed to alter decision‐making among professionals (McGuire et al., 2022).

To understand the challenges that services face in providing support to CEYP, the aim of this project (called the ADaPT project) was to explore what helps and hinders the delivery of cognitive therapy for PTSD in services to CEYP (CT‐PTSD; Smith et al., 2010). CT‐PTSD is a type of tf‐CBT recommended by NICE and developed in the United Kingdom. By exploring the facilitators and barriers of implementing CT‐PTSD, we hoped to understand what support professionals and services may need to be able to provide this best‐evidenced intervention for CEYP experiencing PTSD. To do this, we worked alongside 28 mental health teams across England, including general child and adolescent mental health services (CAMHS), targeted CAMHS (i.e., those specifically for young people in care) and social care‐based mental health teams, conducting focus groups and interviews to explore their experiences of delivering CT‐PTSD with CEYP.

## METHOD

### Research governance

The study protocol was pre‐registered at https://www.isrctn.com/ISRCTN38238325. Ethical approval was provided by HRA (IRAS ID: 307056) and additional approvals were gained from partner Universities and each National Health Services (NHS) Trust and/or local authority.

### Mental health teams and professionals

To be included in the study, teams and professionals were those who endorsed that at least part of their role involved providing direct mental health support to CEYP. Using existing networks and snowball recruitment, with consideration of geographical spread, we approached social care and NHS mental health teams across England. Three NHS child and adolescent mental health (CAMHS) teams declined participation due to lack of capacity to be involved in research or to prioritize the target treatment. We did not collect any further information on teams who declined. In total, we recruited 28 mental health teams, across 14 geographical localities across the North, East, South‐West and South Coast of England, and Greater London. They spanned 11 NHS Trusts and three local authorities. Of these 28 teams, we trained 24 in CT‐PTSD, and 4 were already trained (in either trauma‐focused CBT [Cohen et al., 2012] or CT‐PTSD [Smith et al., 2010]). At the time of recruitment, 11 teams were general CAMHS, 9 were targeted CAMHS specifically for young people in care (formally NHS but often embedded in social care), 4 were specialist outpatient CAMHS (not exclusively for young people in care), 3 were social care‐based mental health teams and 1 was an inpatient team. Twenty‐six of the 28 teams provided data reported in this paper (via qualitative interviews/focus groups). Of the two teams who provided no qualitative data, one was an already‐trained general CAMHS (*n* = 5 professionals) and one was a specialist CAMHS rapid response team (*n* = 4 professionals). For those we trained, we were able to compare sites of those who implemented versus those who did not, while those already trained provided useful additional information on their implementation process.

In total, there were 243 participating mental health professionals who consented to the project. Of these, 196 (80.7%) were trained in CT‐PTSD as part of this project. Of the remaining 47 participants, 29 were in teams already trained in a tf‐CBT and 18 were not trained. This latter group were team managers/leadership or assistant psychologists or assessors, who provided input on implementation issues but were not involved in treatment delivery. Early in the project, one social care team formally withdrew from the study (*n* = 22 professionals) as leadership decided to revisit whether they should offer direct mental health interventions. They provided an exit qualitative interview to allow their feedback to remain incorporated into the project. Beyond the 22 participants from this team, a further 38 participants withdrew over the 18 months of the project. The most common reasons for withdrawal from these 38 participants were as follows: moving teams (*n* = 29, 76%) and being on extended leave (e.g., parental leave and sickness leave; *n* = 7, 18%).

### Intervention training and additional implementation support

Teams completed a two‐day training in CT‐PTSD (Smith et al., 2010). Within this training they were also introduced to the Child Revised Impact of Events Scale (CRIES‐8; Perrin et al., 2005), as a way to screen for PTSD symptoms in their young people. Trainings were run both in‐person and virtually, all led by expert clinicians. CT‐PTSD is formulation driven and usually delivered over 10–15 sessions, although more sessions may be expected for complex cases (NICE, 2018). CT‐PTSD contains core elements of a tf‐CBT treatment, including psychoeducation, the development of a trauma narrative (using a timeline approach for multiple exposures), updating the narrative, working with triggers and reclaiming life.

Participating teams had access to a variety of optional implementation support across the project. This included top‐up training sessions, bi‐monthly virtual supervision drop‐in sessions, webinars on complex case formulation and working with complexity, and access to animations and training videos developed as part of this project (the animations and training videos are freely available at uktraumacouncil.org; details provided in Supporting Information).

### Data collection

Following training (or from recruitment for those previously trained), teams were followed up for 12–18 months. Over this time, participants took part in rolling focus groups with their team, approximately every 3‐months. Focus groups were semi‐structured to gain insight into views on the implementation of the intervention, with a focus on facilitators and barriers. Where teams worked closely in the same locality, all would usually attend the focus group together (e.g., the assessment team, the treatment team and the inpatient team). Focus groups were mostly facilitated using MS Teams and were recorded, with occasional focus groups conducted in‐person. Focus groups were mostly 1 hr but ranged from 30 to 90 min. There was also the option of completing a 1:1 interview over the phone or via MS Teams for participants who could not/did not want to attend scheduled focus groups. Besides the individual team/locality focus groups, we also ran two cross‐locality focus groups with the team managers and clinical leadership. Finally, all researchers on the project worked closely with multiple teams, providing useful insight into the culture and relationships within and between teams. To capture this, researchers made ethnographic field notes.

In total, we ran 58 focus groups and 62 individual interviews. At the end of each focus group or interview, the interviewer (trained research assistants/associates) completed an interview summary report, which was a two‐page document that summarized the key discussion points. These were broken down into three sections: facilitators of intervention implementation, barriers to implementation (and strategies that may have been trialled to address barriers), and other key insights (e.g., about the team and broader environment). All summary reports were quality checked against the full interviews, by a different researcher to the interviewer. This quality checking also involved cross‐checking for any missing information, clarifying audio/transcripts and identifying quotations. Data were also collected on the use of the CRIES‐8 PTSD screening tool, as well as some quantitative data exploring implementation and service pathways, which will be reported elsewhere.

### Data analysis

Data from the summary reports were analysed using a framework analysis. This was conducted using the updated Consolidated Framework for Implementation Research (CFIR 2.0; Damschroder et al., 2022). The CFIR 2.0 is a determinant framework offering a comprehensive, organizing taxonomy of operationally defined constructs that may impact the implementation success of complex programmes and an overarching typology that helps to identify what practices work within different contexts. Two members of the team (RM and DS) coded the summary reports using this framework. In an initial training phase, both independently coded four reports, followed by a consensus meeting with senior author RH and CFIR expert KH. Following this, RM and DS each coded 50% of all reports, meeting weekly to discuss the coding framework, where some original CFIR codes were modified to better reflect the data. Once all summary reports were coded, RM, RH and DS met to generate themes based on the individual CFIR codes. As there were many references under each code, we decided to merge codes to create key themes, rather than write about each CFIR construct individually.

Ultimately, three CFIR domains were included: outer setting, inner setting and individuals. The outer setting domain refers to the macro‐level factors that influence and can be influenced by the inner setting and individuals within it. The inner setting refers to characteristics of the setting in which the intervention is implemented, that is, the mental health teams. The individuals domain refers to the roles and characteristics of individuals that can affect implementation. Codes from the intervention characteristics and implementation process domains were either merged into these themes where relevant or will be presented elsewhere. While themes are presented separately, they are interrelated. They include discussion of universal barriers (i.e., those that impacted most/all teams) and unique barriers (those that differentiated teams who did and did not implement CT‐PTSD). Quotations related to themes and subthemes are embedded throughout, with further examples provided in Table S1.

As a final step, we drew on ethnographic field notes provided by the researchers who supported each team to further refine themes and add descriptive information.

## RESULTS

### Sample descriptive statistics

Full descriptives for the sample are presented in Table 1. Professionals were mostly female (87%) and White (91%). The largest professional groups were clinical psychologists or trainee clinical psychologists (29%), social workers (22%) and mental health nurses (19%). Participants ranged from trainees or newly qualified to those with 37 years of experience in their profession.

**TABLE 1:** Description of sample (*N* = 243).

Gender, woman (*n* = 232) *n* (%)	201 (87%)
Ethnicity (*n* = 231) *n* (%)	
White	210 (91%)
Asian	6 (3%)
Black	5 (2%)
Mixed ethnicity	6 (3%)
Another ethnicity	4 (2%)
Age range (*n* = 231) *n* (%)	
18–29 years	26 (11%)
30–39	84 (36%)
40–49	67 (29%)
50–59	45 (20%)
60+	7 (3%)
Profession (*n* = 233) *n* (%)	
Clinical psychologist (including trainees; *n* = 5)	67 (29%)
Social worker	51 (22%)
Mental health nurse	44 (19%)
Psychotherapist or creative‐based therapist	21 (9%)
Clinical associate psychologist	9 (4%)
Occupational therapist	6 (3%)
Counsellor	6 (3%)
Assistant psychologist	3 (1%)
Other^a^	25 (10%)
Years qualified (*n* = 223), *M* (*SD*)	10.2 years (8.7)

^a^ ‘Other’ professions include CBT therapists, psychological well‐being practitioners, personal advisors, and practitioners/specialists working in the community, residential care or education.

### Overall implementation

Overall, 46% (11/24) of the trained teams were able to implement CT‐PTSD, although only 25% (6/24) were able to implement it with CEYP specifically. At least one team was able to implement in 57% (8/14) of the participating geographical localities, and 43% (6/14) of the localities had at least one team able to implement CEYP specifically. Of the targeted children in care‐specific NHS teams, 33% (3/9) were able to implement the treatment. A summary of the unique and universal implementation themes are presented in Table 2.

**TABLE 2:** Overview of themes.

CFIR domain	Overall theme	Summary	Unique or universal^a^
Outer	Service structure and relationships between sectors/services	Across the country, different regions had very different service set‐ups, which impacted on their ability to implement the intervention. Most specialist mental health teams for children in care were unable to implement because they were commissioned to work with the network and did not have additional capacity to provide direct psychotherapy. Some services could provide some direct support, but only for a short number of sessions (and therefore could not accommodate CT‐PTSD). Care‐experienced young people were also often not referred to services that might provide this type of direct intervention (or referrals may have been rejected). This meant that in many regions, commissioning practices and pathways between services meant young people in care would struggle to access direct NICE‐recommended psychotherapy of any kind.	Unique
Outer	Characteristics of the environment around the young person	Across all sites, regardless of ability to implement, the characteristics of the young person's environment were considered central to the potential to both start and successfully complete the intervention. Many professionals discussed the challenges of delivering the intervention to young people where there was a lot of instability in the environment (e.g., placement instability and school refusal). Many saw caregiver involvement as important in facilitating the delivery of the treatment and ensuring the young person was supported, but for some young people (especially adolescents) there was no available or appropriate caregiver	Universal
Inner	Supervision, support and leadership style	Having engaged and supportive leadership, who advocated for CBT and prioritized supervision, was crucial for implementation. They were able to hold therapists' anxiety about the treatment but still support them to implement. All teams that implemented had strong supervision structures, which provided safe spaces specifically to discuss the use of trauma‐focused CBT	Unique
Inner	Team culture, buy‐in and prioritization of decisions	Across teams, there were different cultures and belief systems around the suitability of CBT treatments (and trauma‐focused work) for care‐experienced young people, and of the use of diagnostic labels like PTSD. Sometimes these concerns were also reflected in the team's implementation engagement, where the team might also be wary or cynical of the research. In contrast, some teams used the project as an opportunity to bridge gaps between mental health and social care teams and consider how their structures worked for care‐experienced young people	Unique
Individual	Complexity of young people and therapist perception of readiness for treatment	The complexity of the young person's presentation was a universal challenge discussed by all services. Care‐experienced young people referred for mental health support often experience a range of other complexities, including placement instability and risk or safeguarding concerns. Within and between teams, there were individual differences in how professionals approached this, reflecting a unique implementation driver. Sometimes, these complexities and hesitancy from young people were taken to mean the young person was not ready for the treatment, whereas some saw this as a standard part of working with PTSD/complex PTSD. Those who took the latter approach often were in teams where the outer and inner factors were also in place to support implementation.	Unique
Individual	Mental health professionals' capability, confidence and willingness	It was quite normal for professionals to express worries and lower confidence around delivering the treatment. Expressing this was not necessarily a barrier. Indeed, within the context of supportive teams, those who could openly reflect on their anxieties and their own potential avoidance were generally those who were able to implement. However, if professionals were unable to see cases quite soon after training, and if there were not strong CBT advocates whom they could go to for confident support, implementation was difficult. Additionally, for some professionals, CBT did not align with their therapeutic approach or their beliefs, which meant they did not implement	Unique

*Note*: CFIR is the consolidated framework for implementation research (discussed in Methods).

^a^ Unique implementation barriers are those that differentiate implementing versus non‐implementing sites. Universal barriers are those experienced by all sites.

### Outer setting

Within this domain, key factors affecting implementation were twofold: (1) the commissioning and set‐up within and between services and (2) the environment/network around young people.

### Theme 1: Service structure and relationships between sectors/services

#### Subtheme 1: Commissioning and capacity

There were substantial differences in how services were set‐up in different regions, particularly in terms of the types of services accessible to young people in care. Most targeted CAMHS teams (teams specifically or predominantly for children in care) were more closely embedded in social care, rather than general CAMHS, although in some regions the opposite was true. In some regions, there were teams that comprised a full targeted service for young people in care, while in others, there were individual therapists who provided this targeted support, but they were embedded in a wider general CAMHS team. In some regions, it was unclear whether there was a targeted CAMHS team at all.

There were several discussions among professionals where they voiced their frustrations about the regional differences in referrals, eligibility criteria and the type of work offered. This was thought to have a unique impact on young people in care, as they may be moved around different placements or out of area for a particular service.

…different CAMHS services in different boroughs having different rules about who sees the kids, it just drives me potty… these are the most vulnerable people and yet they're the ones that are moving around and that means that their mental health treatment has to move with them… But that's the way that services are commissioned and set up

The overall view was that an ideal system or service model would allow for both work with the network of professionals and carers involved in the young person's life (e.g., consultation and carer support) and individual psychotherapy with the young person themselves. However, this level of service was rarely available either within or between teams. Across all regions and teams, participants reported insufficient resources (primarily staffing) to address the large number of young people who might have benefited from CT‐PTSD (or indeed direct psychotherapy for a range of issues). Services were also viewed as often unable to provide the time/resource to allow professionals to deliver the complete intervention (e.g., only being able to offer 6 direct therapy sessions, rather than the 10–15+ recommended by NICE).

We're restricted in what we do because we have to show that we're saving money… We don't have enough resources to bring enough people into the team so you're a team that does a little bit of everything because you don't have enough funding that's prioritised

These pressures were often perceived to have a particular impact on ability to deliver CT‐PTSD. Some felt they simply did not have capacity or resources to learn a new therapy and adequately prepare for a session, let alone attend/provide supervision.

Understanding the pressure there is of bringing in a new model into an existing system and how much support and how many additional hours need to be agreed and protected if practitioners are going to do a good job

#### Subtheme 2: Ability to deliver individual psychotherapy and contact with young people in care

A prominent finding was that many targeted children in care teams were unable to deliver individual psychotherapy (including CT‐PTSD), or that this happened very rarely. For these teams, they were primarily commissioned to support the network around the young person. Thus, despite entering the project due to a belief that they could deliver the intervention, in practice this was near impossible for many teams.

And actually it [being in the trial] did make us realise that we very rarely offer direct individual therapy

In addition, many general CAMHS teams were able to implement the intervention, but not to young people in care simply because they did not see any/many. In many regions, young people in care seemed to stay within targeted or social care mental health teams, where they would often not receive individual psychotherapy. However, they were also not referred, or not accepted, to general CAMHS, even where they presented with high PTSD symptoms. Overall, in many regions, it was apparent that young people in care were falling through the gaps due to specialist services not delivering this direct psychotherapy, but young people in care not being referred to general CAMHS services (or having referrals rejected). Many services were unable to match intervention to the individual needs of the young person, or to deliver a direct intervention at all, even if the professional recognized that it was needed.

They say that there is a pressure to be a good clinician – between being a good clinician on paper and do what you're governed and funded to do – and be a good clinician by doing what is really best for the young person

While only a small number of services in this project were fully social care based, it was apparent that these services faced particular challenges when deciding what to offer or what the role of their team would be within the wider mental health landscape. Some of these services were relatively new (compared to NHS Trust teams), and still navigating whether or not their team should include direct diagnosis‐ or needs‐driven interventions, like CT‐PTSD. One social care team signed‐up for the trial and later withdrew on the leadership decision that they would no longer identify as a mental health service, despite holding a substantial amount of responsibility for the emotional well‐being of young people in care. Another social care team were able to implement, and had structures that meant the team could offer both network work and direct psychotherapies.

We are not a mental health service, we are a service to support young people with [placement] stability

#### Subtheme 3: Relationships and pressures between sectors

How services and sectors worked together (or not) was key to implementation. Having open communication and positive relationships with social workers and social care staff was an important facilitator of CT‐PTSD delivery. Where there was miscommunication, it could result in blurred boundaries between roles, which affected the ability of mental health teams to implement the treatment. However, where there were strong links and respect between sectors (particularly social care and mental health), professionals were able to come together to establish different roles to support the young person through the intervention. This included making specific time to discuss cases with social care colleagues.

The real big pro is to actually be close to the social workers to be able to really connect and catch up with them and… to be able to foster those relationships closely

In contrast to work practices that supported implementation, some professionals discussed challenges in deciding whether to deliver CT‐PTSD when there were different opinions within their teams and from other sectors. This was particularly apparent in discussions around ongoing court cases or care proceedings, where there would often be pressure from law enforcement or social services to not begin or to stop trauma‐focused work while the proceedings were ongoing.

I'm perhaps hearing mixed different kind of views [about when to deliver CT‐PTSD] from what we received in the training compared to what I'm hearing from other places and other people and other teams. And so, there's, I guess, concerns being raised actually is that appropriate

### Theme 2: Characteristics of the environment around the young person

Across all teams, regardless of ability to implement the treatment, the characteristics of the young person's environment were considered central to the potential to both start and successfully complete the intervention. This reflected a universal implementation barrier, in that it was an issue all teams grappled with.

#### Subtheme 1: Environmental (in)stability

A primary perceived barrier to using the intervention was the instability of the young person's network and general environment. Many professionals reported that they often were responding to acute crises or safeguarding issues, which meant they were unable to consider starting direct psychotherapy. Again, implementation here was facilitated by working closely with social care colleagues to ensure all understood the treatment plan and discussions could be had (for example) around who was responsible for safeguarding and risk, to allow the mental health professional to focus on treatment delivery.

You know, you sort of need a case manager doing all of the containment, you need somebody doing the systemic and family work or care or consultation, and then you need somebody with permission to really focus on trauma and not to have their entire one hour a week opportunity taken up with safeguarding

There was also often concern about the stability of the young person's support network, which made it difficult for professionals to judge whether or not to start the intervention. Sometimes, there was particular concern that placement changes could move a young person out‐of‐area region. This raised various concerns, including that there might not be a safe adult to support them through treatment; as well as practical concerns like an out‐of‐area change making a young person ineligible for the service; or an upcoming 18th birthday meaning the young person may become ineligible.

Is it appropriate for this client population when this [placement breakdown] can happen at any point of time, and how do you then sort of pull back and contain that young person, especially if they've been moved out of borough and into a different service

Instability was also apparent in the professional network, which many discussed as a barrier to both assessment and intervention. Young people were often coming to services and professionals with long histories of difficult interactions with professionals (whether legal services, social care, education, other mental health services, etc.). These experiences could affect the young person's perceived willingness to engage in treatment.

And then they've met so many social workers when they first got here, and often they've had so many conversations, been promised things and nothing happened. And then I'm just another professional in this long line of, you know, sometimes quite disappointing encounters and quite intrusive encounters… it's a hard one because they have to come to the same place where they would meet their social workers to meet us…

The challenges of an unstable environment were discussed by all teams. However, teams that were able to implement more often reported finding ways to deliver the intervention in the face of instability, either by delivering discrete stabilization sessions (e.g., emotion regulation) as a way to then move on to core components CT‐PTSD, or ensuring sessions could hold time for both stabilization strategies and CT‐PTSD, even when young people come in with a recent crisis (discussed further later).

#### Subtheme 2: Caregiver support and involvement in therapy

Many professionals discussed caregiver involvement as a facilitator of treatment. This was not only due to their importance in supporting the young person but also because of views that the network might also benefit from the additional information about the young person's needs, to develop their understanding and ability to support young people in their day‐to‐day.

Where caregivers were able to provide support and advocate for their young person, professionals felt more able to implement the treatment. Relatedly, where caregivers or another support person (e.g., social worker) was not available, or actively discouraged/blocked access to CT‐PTSD due to their own views about the young person's stability/readiness, there was often hesitance from mental health professionals about starting CT‐PTSD – particularly given it would involve discussing trauma, due to concern that the young person may require on‐going support after the session. Sometimes there was no caregiver due to the placement type, instability of the network, and/or the young person specifically not wishing an adult to be involved. In other cases, it was due to logistical issues, such as the carer having other caregiving commitments or the social worker having work capacity issues.

It's that argument of kind of like how stable does their support network need to be? And I'm I guess I'm on the fence, really. I sort of see both sides of it. However, I do sit with, if a young person doesn't have that support outside of a therapeutic session, they're kind of left sitting with that

### Inner setting

Most teams struggled with outer setting issues. Yet, even in the context of complexity, commissioning issues, and service constraints, many teams were able to implement CT‐PTSD. A major differentiating factor between whether or not a team implemented the intervention was the inner setting, which covered (1) how the team were supported by leadership in treatment delivery; and (2) team buy‐in and prioritization of treatment delivery.

### Theme 1: Supervision, support and leadership style

Team leads were key figures who could help or hinder implementation. Leads who were well liked and respected and often had a background in CBT (and therefore, confidence in CBT) were able to scaffold the team's learning and build confidence.

[NAME] who is our clinical nurse specialist and sits within the leadership team – they're one of the CBT therapists – is very passionate about this work anyway. I think that rubs off, so they'll have conversations with people “well have you thought about trauma focused CBT?” cause they're in that role to influence a little bit

These team leads were also able to recognize and acknowledge the anxiety or worry that some professionals may have felt when starting out with the treatment, and support their learning and use of the intervention.

What I find is, after they've had the first successful case, they're sold on it. So it's getting them over that first case of getting somebody to do the narrative … it's really tricky for everybody, almost whatever their background, even if they're really bought into it. They understand CBT, they're still unsure until they've had a success

However, leadership buy‐in alone was often not enough. For example, if the culture of the wider team was ‘anti‐CBT’, even very committed leaders could struggle to motivate a team to implement. Furthermore, leadership can change, so having only one person to advocate creates a single point of failure. A professional discussing a strong CT‐PTSD advocate and source of support being on long‐term leave from the service noted:

I do feel like there was a bit of a void, actually, because she would be, she's very knowledgeable, like member of staff, she is amazing and she would probably be the one that we would go to for the supervision

Leadership is often linked to another crucial aspect of implementation – supervision. It followed that those who implemented in their service were also those who established supervision structures. These supervision structures were often new and specific to PTSD/CT‐PTSD, to respond to the team's needs. This also reflects a prioritizing of supervision time within the team and by leadership. It also meant, among the busyness of services, that CT‐PTSD was ‘kept on the table’.

This is a really nice work environment… We really love working here and it is a naturally very supportive team I would say. And we do have a specific monthly trauma focused CBT supervision group now thanks to this trial. So we bring our cases that we're thinking about trauma focused CBT to that group, but we also have weekly case discussion slots

In contrast, there were examples where the team were motivated to deliver the intervention, but leadership did not support the set‐up of supervision groups and did not seem to actively encourage delivery. While some of these teams attempted implementation, it was usually short lived without the leadership support and prioritization of supervision time. There were some examples of leadership actively discouraging the use of the intervention, in particular, with young people in care (but supporting use with other young people). In general, a lack of support from leadership meant professionals were left feeling unsupported and ultimately were less likely to implement.

And from a management point of view, was there support? I mean, I think it was good that we were able to access the training but again was there a bit of a gap between management level and us actually delivering it

### Theme 2: Team culture, buy‐in and prioritization of decisions

Overall, a key factor differentiating whether or not a team implemented was the whole team's buy‐in to the CT‐PTSD model, which also often related to their engagement with the implementation process and their engagement with PTSD screening. Put simply, some teams did not believe in diagnoses like PTSD or in CBT‐based treatments or felt trauma‐focused CBT treatments were inappropriate. This hesitancy was almost always specific to young people in care and the belief that their complexities meant the approach was unsuitable or less suitable than alternatives (e.g., network focused work; more general psychotherapy or creative‐based therapy).

In general, teams who were more motivated to screen for PTSD and deliver the treatment were also those who were generally more engaged in the implementation process. There were multiple examples, across all service types, where teams used the project as a chance to take stock of their current practice, from how they do and record assessments, through to intervention offers and pathways to care. Some used it as an opportunity to reflect on how they worked with their social care colleagues. In contrast, some teams were very uncomfortable and defensive towards the idea that they were being ‘evaluated’. They felt that their involvement in the trial meant that CT‐PTSD was ‘put onto them’ by management/the research team, which negatively influenced implementation.

#### Subtheme 1: Stabilization and the meaning of individual therapy

A major differentiating factor for implementation was how teams viewed and addressed stabilization. Stabilization refers to the process (i.e., sessions) that usually focuses on risk reduction and distress tolerance/emotion regulation. While almost all professionals reported that they felt some stabilization was necessary given the complex needs of many CEYP, how the teams approached this differed, particularly in terms of getting ‘stuck’ in stabilization versus moving forward with key treatment aspects – particularly working on trauma memories. Those teams that were unable to implement often raised the issue of stabilization over multiple focus groups, with little resolution (e.g., plan).

We keep having the same discussions in the team and maybe not getting further with them around the period of stabilisation that we have and use, and I think everyone feels very confident in stabilisation, I think it's something that people do regularly and have done regularly for a very long time. And there's really mixed views maybe within the team around how long that should be

In contrast, teams who did implement often raised stabilization initially but then internally developed strategies to manage this and move forward with treatment. For example, they incorporated stabilization strategies alongside the memory work, or introduced set sessions around stabilization, but kept their focus on moving on to the full treatment.

… sometimes the systems‐work and the hierarchy of needs scuppers you in your efforts of individual care… because PTSD treatment works and we're not getting there because we are a little bit um‐ it's sort of immobilised by some of the complexity… I feel like since we've been exploring all of that… I think we we're doing quite organised work with some good outcomes with PTSD, with some real complex kids.

Beyond stabilization, in some sites, from those who struggled to implement, there were also some concerns expressed that offering any individual psychotherapy to the child might send the message that the child was to blame for their mental health. This was framed around the importance of the network understanding the needs of the child, but could then clearly act as a barrier to implementation of any direct psychotherapy.

It's really, really important to us not to locate the difficulty within the child… we have to think incredibly carefully about how we present the intervention to the network in a way that really reinforces that this is an understandable response to the child's history, and not because there's anything wrong with the child or the way they're responding…

#### Subtheme 2: Competing therapies

Some teams were also offering EMDR. Sometimes this worked well, providing young people with treatment options. However, there were also some examples where senior EMDR therapists would actively discourage the use of trauma‐focused CBTs, and express to other professionals that it would ‘re‐traumatize’ young people. For teams trying to deliver CT‐PTSD, this could be very unsettling. Overall, despite clear guidance outlined by NICE, there was often confusion about how trauma‐focused CBTs and EMDR should be presented and used in practice, and it was also often unclear how teams were presenting this to young people. However, these issues could be overcome by teams being willing to create shared spaces for learning together.

We are setting up some joint time with the EMDR clinicians and the trauma‐focused CBT clinicians… basically somebody will present a trauma‐focused CBT case and somebody will present an EMDR case so that we can understand how the therapies work and then… hopefully get a better understanding of what young person would suit what kind of therapy and that will fit into like the trauma pathway that's being developed as well.

### Individuals

As well as broader factors around the team and environment (outer setting), and factors within the mental health team as a whole (inner setting), there were also some individual‐level characteristics that affected whether the treatment (CT‐PTSD) was implemented. These were mainly focused around the young person receiving treatment and the professional delivering treatment.

### Theme 1: Complexity of young people and perception of readiness for treatment

Along with the environment and network around the young person, some professionals described several other characteristics of the individual young people that meant they were sometimes unsure of whether to deliver CT‐PTSD (or felt CT‐PTSD would be inappropriate). They often discussed the complexity of young people coming through their service, particularly in teams working solely with young people in care (e.g., targeted CAMHS teams). Interestingly, many in general CAMHS would highlight that most young people they saw, including those in care, had complex needs, so were perhaps less likely to see this as a reason not to offer treatment. The level of complexity CEYP present with is consistent across the country; however, it seemed that the teams who did not implement CT‐PTSD referred to this more often as a barrier than teams who did manage to implement. Often those struggling to implement referred to ‘firefighting’ co‐morbidities or crises in the environment.

Usually, whatever happened in school that day, or whichever teachers were mean to her or her GCSEs, like, there's enough stuff happening in the present that we're just trying to get through that

Many of these professionals described not knowing where to start or how to move onto trauma work when faced with young people bringing so much complexity. However, some were able to move past these barriers and deliver CT‐PTSD.

This quiet unassuming child, who is also in the middle of transferring schools and other things so has quite a lot going on for him, has really opened up about how angry he is about the trauma… it's like he's found his voice

Some also referred to young people's reluctance or inability to engage in CT‐PTSD, either to get started or to keep engagement during treatment. Related to ‘Outer settings’, services were often unable to accommodate young people potentially disengaging and re‐engaging later. The issue of young person engagement was raised by many professionals across all service types, but more so by teams who were unable to implement. Reasons for ‘readiness’ were interpreted in a range of ways, with some referring to stages of child development, particular skills (e.g., emotion regulation), or the child's overall motivation or engagement with the intervention (see Table S1 for quotes). However, professionals who were able to deliver CT‐PTSD acknowledged that it was possible to address these barriers by helping young people develop their skills or reduce their avoidance, in order for the treatment to take place. This meant considering the pace of delivery as one possible strategy to maintain engagement, which required services to be able to provide more or longer sessions (which is in line with NICE recommendations). Some professionals, particularly those who implemented, were also able to reflect on their own potential avoidance (see section below for further detail on individual therapist factors).

It's been kind of a balance of I suppose‐ pushing forward with exposure work, but also being aware of how overwhelming that is, particularly for [young person] and thinking about how we balance that in terms of making sure [young person] is still engaged whilst also kind of working towards [young person's] goals as well… the challenge of lots of the trauma work and finding that balance of not kind of colluding with the avoidance but also going at a pace that feels comfortable for the young person as well.

### Theme 2: Mental health professionals' capability, confidence and willingness

Related to young people's complexity, many professionals felt uncertain about how to get started when working with CEYP that present with such complexity.

I've got a case where I want to say I'm contemplating thinking about doing trauma focused CBT but I'm really hesitant, I guess at the moment because it would be the first time that I've delivered it. But this young person is really, really complicated and has recently disclosed another trauma… so it's a really potentially complicated one to start with

Many also acknowledged that if they were not able to get started soon after the training, this resulted in reduced confidence, as they struggled to remember and consolidate their learning.

I'm not sure how, how confident I would feel going into a new piece of work. I think because so much time has passed since the training and where the first one didn't massively get kind of up and running it, it feels like that almost that that window where I would have been able to consolidate perhaps some of the training and go back and refresh myself. It feels like so much time has passed.

Some professionals acknowledged that while they can get started, it can be more difficult to move on to the more intensive elements of the therapy (e.g., memory work). Often they would discuss this as relating to the child's avoidance. Some teams/leaders were able to recognize and discuss the potential that therapists themselves might engage in avoidance. As discussed earlier, team cultures that enabled open and non‐judgmental conversations about individual concerns (even via informal peer supervision) were generally more able to implement.

Often we don't get the reliving work… That's an end point for anything, but I know that that's been some of the discussions we've had as a team. Is that actually, is this our avoidance? Is this their avoidance?

Other professionals who did not deliver CT‐PTSD stated that this was not due to confidence or anxiety but ultimately because they did not agree with the therapeutic approach. This appeared more common in services specific for young people in care, where some felt that trauma should be addressed or processed in a different way. These individuals did not implement, regardless of whether the wider team were implementing.

Less about confidence and maybe more in alignment to different models that feel more helpful for a population. I think for me in my trauma toolbox, tf‐CBT would be the last one I'd think of using for a lot of reasons…I think others are more creative, I think they're less cognitive, I think you can be more flexible in the treatment approach in thinking about the young people who do present with a trauma presentation. I think those are my main reasons.

## DISCUSSION

The primary goal of this project was to work alongside mental health professionals to understand what helps and hinders the delivery of NICE‐recommended treatment for PTSD to CEYP. Findings showed important barriers in the outer settings that particularly impacted on the ability for targeted services to implement (i.e., service structures/commissioning/resources), as well as the universal challenge of delivering CT‐PTSD to young people with very complex needs. Unique barriers that differentiated implementing from non‐implementing teams were particularly found in inner settings, related to leadership, culture and team beliefs. Taken together, the findings provide insight into what mental health teams, service leads, commissioners and policy‐makers need to consider if services are to deliver best‐evidenced practice to children in care and CEYP more broadly. Table 3 provides a brief overview of key recommendations.

**TABLE 3:** Key recommendations for the implementation of trauma‐focused CBTs.

Target audience	CFIR theme	Recommendation
Commissioners	Outer setting	Commissioners should be aware of evidence‐based practice and ensure there is specific commissioning for the delivery of NICE‐recommended needs‐matched treatments
Commissioners and Service leadership	Outer setting	Relevant social care and mental health staff should prioritize leadership and commissioning meetings to ensure pathways between sectors mean a young person in care could access direct needs‐matched psychotherapy
Service leadership	Outer and Inner setting	Commissioning and leadership should include a prioritization of high‐quality assessment practices that use standardized assessment and screening tools, allowing treatment to match needs
Service leadership	Outer and Inner setting	Leadership should prioritize in‐house supervision structures that are specific to evidence‐based trauma‐focused mental health treatments
Trainers and Service leadership	Inner setting	When training professionals, trainers should work with leadership to ensure teams have appropriate clinical cases to get started on as soon as possible after training. Training should include a specific focus on working with complexity and how to not get ‘stuck’ in stabilization
Service leadership	Inner and Individual setting	It is important for leadership to invest in staff development and competency development, including prioritizing clinical professional development opportunities. Relatedly, ensuring there are CBT advocates who are confident in delivery of CBT‐based approaches is likely important for implementing CT‐PTSD
Service leadership and team	Inner and Individual setting	It is important that teams recognize and discuss their own potential biases that may prevent the delivery of best‐evidenced needs‐matched mental health interventions, which can particularly affect young people in care

A challenge for many teams in the project was the lack of adequate resources to deliver CT‐PTSD. The under‐resourcing of CAMHS is not a new issue (e.g., see Atkinson et al., 2007; England & Mughal, 2019), nor is the under‐resourcing of children's social care (Kerfoot et al., 2004). The consequences of this are long waitlists and wait times, challenges in accessing services for young people and challenges in delivering care for services, with consequences for young people, carers and professionals. These constraints also likely contribute to the inflexibility that we found acted as a barrier to implementation. This includes losing access to services when moved out of area (even if partway through treatment), the inflexibility around length of sessions or the ability to easily re‐engage after a period of disengagement, and the discontinuation of services at 18 years of age. These are all likely to be issues that have a particularly detrimental effect on young people in care. Young people in care are less likely to have a consistent adult advocate and those with the greatest mental health needs are also more likely to be in unstable placements (Hiller et al., 2023; Newton et al., 2000), which may influence their ability to engage in treatment. In the United Kingdom, rates of young people in care being placed ‘out‐of‐area’ also continue to increase (Foster, 2021) and these are most likely to be older teens in particular need of mental health support, and at risk of homelessness when they ‘age out’ of care. This age coincides with many CAMHS discontinuing treatment and young people needing to move to adult mental health services, with the major challenges and lack of appropriate processes and support during this service transition well‐documented (Belling et al., 2014). Policies such as raising the age cut‐off to 25 years are crucial targets, but can only be properly implemented where funding and capacity allow. There is also an urgent need to develop a stronger evidence base for supporting young people in care through treatment when there is no consistent adult support. Many clinicians voiced a lack of caregiver and/or social worker involvement as an issue. Meta‐analytic review has shown that caregiver involvement in treatment does not significantly predict effectiveness (de Haan et al., 2024). However, it seems likely that it would be beneficial to  have a supportive or engaged adult at home (even if they do not directly attend treatment sessions).

Across the services in our study, many general CAMHS teams reported that their involvement in the project had highlighted to them that they saw very few young people in care. While practice differed substantially between regions, young people in care seemed to largely be held at the targeted or specialist NHS CAMHS level or social care mental health team level. Yet, many of these teams were unable to deliver CT‐PTSD primarily because they were not delivering individual psychotherapy. Thus, in general CAMHS, the implementation challenges to CEYP primarily stemmed from a lack of opportunity (not seeing young people in care), while in targeted or specialist teams, it was a lack of capacity and commissioning targets. Targeted services predominantly focused on the network around the young person (e.g., via social worker or caregiver consultation and training). This work is crucial to ensure the carer network is supported in their roles, both to retain/stabilize placements and reduce stress (Adams et al., 2018), as well as for the potential positive influence on the young person (Schoemaker et al., 2020; Solomon et al., 2017). However, it has also been documented that foster carer support alone is often insufficient for meeting the needs of young people experiencing high rates of psychopathology (Minnis et al., 2001; Schoemaker et al., 2020) and carers have voiced frustration at the perceived inadequacy of access to direct therapeutic support for the young people in their care (Hiller et al., 2020). Overall, a key finding was that many young people in care could not access direct NICE‐recommended psychotherapy – CT‐PTSD or otherwise – because of service set‐up. This speaks to the need to review commissioning practices within children in care‐specific services and to better understand resource allocation, referral pathways and how and why therapists may decide whether or not to refer a young person on to general CAMHS (and whether and why general CAMHS may or may not accept the referral).

This project involved two (plus one that was de‐commissioned during the project) social care‐specific mental health teams, although many of the children in care‐specific NHS CAMHS teams were closely embedded in social care. While we cannot draw definitive conclusions based on two services, these teams may need particular support to understand what ‘best‐practice’ should look like in terms of mental health support, and ensure this is consistent across the country. Yet, there has been very little research on what ‘best practice’ might be for these teams. Social care‐based mental health teams hold a large amount of mental health needs, but their role in assessing or supporting that mental health – particularly, in the framework of assessment and treatment of diagnosable mental health conditions – is largely unclear. Here, one team withdrew from the study on the service decision that they would not be considered a mental health team that assesses mental health symptoms and delivers individual interventions, while the other service was able to implement routine screening and treatment – providing a demonstration of the different practices between regions. Given well‐documented high rates of common and trauma‐related mental health conditions in young people in care (Ford et al., 2007), it is again crucial that all services consider where such support could be accessed (across sectors) and how decisions are made around assessments and onward referrals.

Beyond outer setting issues, the difference between teams who implemented and those who did not was also partly driven by team culture and beliefs around the use of trauma‐focused and CBT‐based mental health treatments (and general views of NICE guidelines). There was still relatively widespread belief that diagnoses like PTSD were not helpful (or even not applicable) in this population and that CBT‐based treatments were not appropriate treatment options. Yet, there is a large amount of evidence to the contrary showing these treatments to be effective for young people with complex presentations (including complex PTSD), complex trauma histories and specifically with young people in care (Cohen et al., 2004; Dorsey et al., 2014; Ford et al., 2007; Hambrick et al., 2016; Hoppen et al., 2023; Jensen et al., 2022). Terms like ‘developmental trauma’ were often used as a mental health description, without a clear or consistent definition or treatment implication. This may further exacerbate inequities experienced by young people in care, meaning treatment is not being matched to individual needs (McGuire et al., 2022; Woolgar & Scott, 2014). That said, it is also true that children with very complex presentations may be more likely to disengage from treatments, including trauma‐focused CBTs (Wamser‐Nanney & Walker, 2023). Work from the United States has shown that older teens in care are at particular risk of disengagement (Esterer et al., 2023). More efforts are needed to disseminate the evidence base in a way that is useable for clinical services and supports high‐quality treatment delivery – particularly with young people with complex needs. More research is also needed on how best to engage young people with complex needs, who we know are at higher risk of disengaging or having poorer treatment outcomes.

There were many examples, across all service types, of team cultures that enabled implementation. While those teams who implemented included professionals from a wide range of professional backgrounds, they often had CBT advocates within their team who were well respected and liked. These teams were able to more efficiently start using the screening tool to find cases to start treatment with, soon after the training. There was cohesion between the leadership and practitioners, and teams were empowered to develop strategies to overcome barriers, including creating space for further training. A commonality between all services that implemented was that they developed space for supervision specifically focused on PTSD/CT‐PTSD. This ensured that there was space for these discussions, including worries or confidence around the treatment. The latter may be particularly important as an opportunity to bolster perceived confidence and competence, given this may be related to delivery quality and treatment outcomes (Espeleta et al., 2022). Where supportive supervision was not established, it was challenging for CT‐PTSD to ‘stay on the table’, particularly if the team were not overly motivated to deliver the treatment. Successful teams were also often those who worked closely with social care colleagues not only to navigate responsibilities around safeguarding and risk but also to ensure social care staff were aware of treatment plans and reasoning. This meant specifically creating space for cross‐sector meetings, ensuring referral pathways and options for care were clear among all teams, while also providing crucial opportunities for relationship building between professionals. Work out of the United States has shown that the more closely social care and mental health services work together, the better the access to mental health support for young people in care (Bai et al., 2009).

### Strengths and limitations

This study had a number of strengths, including the inclusion of a wide range of mental health teams, across multiple regions in England. There were also participants from a range of professional backgrounds, reflecting the composition of English mental health services. This allowed us to develop a detailed picture of barriers and facilitators of implementation. However, findings should be considered in light of key limitations and considerations. First, this project was focused on barriers and facilitators to implementation but it was outside of scope to collect data on the quality of delivery of CT‐PTSD. This is an important future step. With knowledge of what facilitates the uptake of this treatment, attention might helpfully turn to what facilitates high‐quality delivery of the full treatment and sustainability. Second, this was a qualitative study, which provides rich and nuanced insights into barriers and facilitators. However, we cannot quantitatively conclude that certain types of practice or service set‐up were statistically associated with implementation success. There is relatively mixed/inconsistent evidence for whether certain service structures or therapist qualities might predict treatment outcomes for young people, and this remains an important area for investigation (Ryan et al., 2023).

## CONCLUSIONS

Our findings highlight vast differences in practice between and within regions in relation to mental health support for CEYP, and particularly access to needs‐matched NICE‐recommended mental health care. In many regions, a young person in care with PTSD would be unable to access best‐evidenced treatment because of commissioning practices, resource limitations or restrictive or fragmented pathways between services. Findings highlight the need for commissioners and cross‐sector service leadership to reflect on whether services and pathways in their region are set‐up to allow access to NICE‐recommended psychotherapies, such a trauma‐focused CBTs. Importantly, even in the face of major challenges, many teams implemented the intervention, which often reflected leadership styles, CBT advocates, team buy‐in and a culture of openness and prioritization of team learning, support and development. Within the current climate of underfunding, deciding what to prioritize is incredibly challenging for services. When it comes to the delivery of high‐intensity NICE‐recommended CBT treatments, our findings show the need for service leads and teams to work together to create an environment of support and openness, which prioritizes professional development and supervision, particularly from senior team members who are confident in the delivery of CBT treatments. It would be particularly beneficial for services to work across sectors and openly reflect on potential biases in their system, from referral gate keeping, through to assessments and treatment offers, that might inadvertently act to the detriment of young people in care and broader groups of care‐experienced young people.

## AUTHOR CONTRIBUTIONS

**Rosie McGuire:** Data curation; Formal analysis; Writing—original draft; Writing—review & editing. **Richard Meiser‐Stedman:** Conceptualization; Writing—review & editing; Supervision; Visualization; Resources. **Patrick Smith:** Conceptualization; Visualization; Writing—review & editing; Resources; Supervision. **Davin Schmidt:** Data curation; Formal analysis; Visualization; Project administration; Writing—review & editing. **Gretchen Bjornstad:** Writing—review & editing; Conceptualization; Visualization. **Robyn Bosworth:** Project administration; Writing—review & editing. **Timothy Clarke:** Conceptualization; Visualization; Writing—review & editing. **Joe Coombes:** Project administration; Writing—review & editing. **Emma Geijer Simpson:** Project administration; Writing—review & editing. **Kristian Hudson:** Conceptualization; Formal analysis; Writing—review & editing; Visualization. **Paula Oliveira:** Project administration; Supervision; Writing—review & editing; Visualization. **John Macleod:** Conceptualization; Visualization; Writing—review & editing. **Ruth McGovern:** Conceptualization; Visualization; Writing—review & editing. **Paul Stallard:** Conceptualization; Visualization; Writing—review & editing. **Katie Wood:** Project administration; Writing—review & editing. **Rachel M. Hiller:** Conceptualization; Formal analysis; Visualization; Writing—original draft; Methodology; Investigation; Supervision; Funding acquisition.

## CONFLICT OF INTEREST STATEMENT

Patrick Smith co‐authored the therapy manual for cognitive therapy for PTSD for children and young people, and receives royalties from this publication. No other authors have conflicts of interest to declare.

## Supporting information


Data S1.

## Data Availability

The data that support the findings of this study are available from the corresponding author upon reasonable request.
