# Supplementary material for: Access to best‐evidenced mental health support for care‐experienced young people: Learnings from the implementation of cognitive therapy for PTSD
Source: Br J Clin Psychol. 2024 Jul 16;64(1):63–85. doi: 10.1111/bjc.12471 (PMC11797148; doi:10.1111/bjc.12471)
Supplement: Supplementary file 1 — Data S1. [file BJC-64-63-s001.docx]

**Free Resources**

As part of the ADaPT project, we co-developed with care-experienced young adults, a series of four animations covering: *What is PTSD?; What is trauma-focused CBT?; Coping with scary or distressing memories;* and *Thoughts about accessing mental health support.*

All animations are freely available via the UK Trauma Council website, and are free to use and share under creative commons licensing.

<https://uktraumacouncil.org/resources/trauma-and-ptsd>

Also as part of the ADaPT project, we worked alongside expert clinicians to develop a series of seven training videos to supplement or up-skill professionals using cognitive therapy for PTSD. These seven videos cover key aspects of the treatment and are freely available to use and share under creative commons licensing. These videos can also be found on the UK Trauma Council website.

<https://uktraumacouncil.org/resources/trauma-and-ptsd-working-with-children-and-young-people>

Table S1. *Summary of themes with additional key quotes.*

|  | Summary | Additional Example Quotes |
| --- | --- | --- |
| **Outer setting** |  |  |
| Theme 1: Service structure and relationships between sectors/services | General CAMHS, targeted CAMHS and social care-based mental health teams were involved in this research. These services were commissioned and set-up very differently across different regions, which impacted their ability to deliver CT-PTSD and/or support CEYP more broadly. Where teams were able to provide network support and direct work for CEYP, either within one team or across multiple teams, this was felt to be the best model. Teams also felt they were able to work best if they had a good relationship with other sectors. | |
| *Subtheme 1: Commissioning and capacity* | Services across the country and across sectors are commissioned slightly differently, which affects the type or intensity of support that they are able to offer. All services are also experiencing financial cuts, which further limits their capacity, and therefore the support they are able to offer. | - *“…it’s also about limited resources that when you're a small team and you're offering a certain amount of intervention, if you're just going for the direct intervention, then you quickly run out of space and time to be able to offer consultations… [or] groups where we might be working with foster carers who can help support trauma”.* - *“We're not a purely treatment-based service. Our remit of what we can do and offer is so wide and flexible”.* - *“Having enough staff to do the day job would help because inevitably people are then on very long waiting lists either to get assessment or to get intervention and that means their behaviours become slightly more entrenched by the time we’ve seen them”.* |
| *Subtheme 2: Ability to deliver individual psychotherapy and contact with CLA* | Many targeted CLA teams were not able to deliver individual psychotherapy due to not being commissioned to deliver 1:1 therapy with young people. By contrast, general CAMHS teams were able to implement 1:1 therapy (including CT-PTSD), but often did not have CEYP referred to them, or these young people did not meet their eligibility criteria. CEYP may also be closed to general CAMHS services if already receiving network support from a targeted CAMHS service. | - *“We're often part of a network and sort of providing some sort of level of stabilization, risk management support, support to the young person there and then support to the network, but it doesn't necessarily feel like this is somebody coming for therapy on a weekly basis…”* - *“That’s what CAMHS do, they’ll go- ‘environment’, nothing to do with us … Which I think is actually appropriate…I think CAMHS get a bad rep- and I will sometimes give CAMHS a bad rep because sometimes they’re crap- but I think generally- They say CAMHS are closed- well yeah this kid hasn’t got a mental health need.”* - *“But if they [targeted CAMHS] came to us [general CAMHS] and said can you see them for the therapy, we need to prioritise doing the consultation. But this young person needs therapy. Would you do it? We absolutely would. And we live in the same building. A lot of the time. So that would be very easy. But it's just we they- it doesn't seem to happen very often and it's made me think that I need to go away and find out you know.”* |
| *Subtheme 3: Relationships and pressures between sectors* | The relationships that mental health teams have with other services (particularly social care, but also education and law enforcement), and the pressure put onto them by these services, affected their ability to support young people and to deliver CT-PTSD. Good communication and clear boundaries were important for professionals to understand their roles and responsibilities in supporting the child (which links to the child understanding who to turn to for particular support). | - *“So, I think a lot of the CLA team's time is driven by the needs of the local authority and the social workers and the foster carers. So that's partly why maybe that a lot of what they do is consultative and they do direct work with foster carers a lot that sort and with social workers but they… do less direct work with kids.”* - *“It's really it is a problem and I think school and it's not their fault. I think that they are really under resourced but I guess the education system like I do feel a lot for some of the young people. I see if the education if they had the right support in schools then that like no matter how much therapy I do that's not gonna sort the issues out it's the support that they need in school.”* - *“Even though we felt like there was quite a robust plan in place, you never know what’s going to happen do you when other people, when you’re kind of relying on biological parents and, you know, other systems for that kind of ongoing continuity or post-18 plan.”* |
| Theme 2: Characteristics of the young person’s environment | Across all sites, regardless of ability to implement, the characteristics of the young person’s environment were considered central to the potential to both start and successfully complete the intervention. This reflected a universal implementation barrier, in that it was an issue all services grappled with. | |
| *Subtheme 1: Environmental (in)stability* | A key barrier to delivering CT-PTSD for CEYP was the young person’s environmental instability. MHPs were often unable to engage in direct psychotherapy, including CT-PTSD, due to responding to acute crises or safeguarding issues. There was also often concern about placement stability, particularly around the young person’s transition out of care, which could result in them moving out of the region, so no longer being within reach of the mental health service. | - *“There’s a real high level of placement breakdown, and general instability in the sort of adoption and fostering world…we do think quite carefully about structuring interventions… when we know there’s an imminent placement move or everyone’s saying it feels like the placement is unstable, we would take that in mind when we’re thinking about what we’re addressing with the young person”.* - *“They all score quite highly and they have been exposed to significant trauma but they won't be- almost certainly won’t be- receiving treatment through the service. And that's because again, it's about stability, placements, where they live…”* - *“I think not being able to work with the system in the full network to the extent that we did previously massively impacts. I'm thinking of a few where we haven't, where direct work hasn't been appropriate because the system around them has just been chaos. Whereas previously would have done a lot of that support in the care team, getting everything almost settled, getting that young person to a place where they'd be able to engage in an appointment, and then we'll be thinking about that direct work.”* |
| *Subtheme 2: Caregiver support and involvement in therapy* | MHPs often felt that it was important for the caregivers to be involved in treatment to an extent, to help develop their ability to support and understand trauma. However, this was not always possible for a multitude of reasons. Some MHPs also felt that to do CT-PTSD, it was important for a caregiver (or another professional e.g., social worker) to be able to support the young person outside of therapy sessions. | - *“And I think often, the CRIES-8 [PTSD screening measure] has felt like we're not ready to use it in some of our cases because of that really kind of immature, that very kind of early understanding of what trauma has been experienced and do they understand that themselves, let alone the foster carers or the network around them understanding it and what's been experienced in terms of neglect or abuse, and how you then ask a question like you know how often do you think about it?…”.* - *“A lot of the time it has turned into family work, cause they’ve needed it at sometimes but also she’s [the carer] like ‘when are we going to get to the trauma work’, and no matter how much I say, you know that’s what we’re doing, she’s getting better, she’s talking to you outside the session, she’s bringing stuff up, she’s volunteering information that she didn’t before, it’s still not trauma work in her eyes.”* - *“I think they also have to have a figure- an attachment figure- that is going to be around for them, whether that's you know, whether that's a foster carer or a social worker, you know, somebody that is going to be that person that sort of picks them up after the therapy session, they go home to that’s going to be supportive.”* - *“I think there are some systemic factors it has been- it’s difficult sometimes. Mostly to do with the anxiety that's held by the staff in the residential homes. Who I’d say, you know, if they’re in a residential home they would come to the appointments. They don't always have staff who- you know- you make the decision for the child and say that ‘I don't think she's up to it today or she's had a bad day, bad week’ and yeah, those sorts of issues do get brought to supervision cause they raise the blood pressure of the clinicians.”* |
| **Inner Setting** |  |  |
| Theme 1: Supervision, support, and leadership style | Many MHPs stated that having the right support available was crucial to whether they could implement CT-PTSD. Teams that were able to implement well, had passionate team leads who shared responsibility (to avoid single point of failure) to run 1:1 or group supervision sessions focused on CT-PTSD or trauma more broadly. | - *“Staffing has definitely been an issue and I think supervision…bringing the supervision together, having the- everybody having the capacity to attend supervision at the same time. And then obviously, I suppose one of our professionals that did the training, who was really leading on it and she had a bereavement and she's been off for a little while as well.”* - *“That would help everyone really hold trauma-focused CBT in mind, you know. So whether it's like a regular supervision, you know, everyone has clinical supervision obviously, but it depends on the kind of, yeah, the orientation of the supervisor I think- but yeah also having that collective space maybe would be something that would have increased it.”* - *“I think what I like that obviously we work quite big caseloads and I think it's nice to have like I met with [clinical lead] for some sort of supervision that focused kind of almost like protected time to actually think about the navigation in depth with someone… So having or making that headspace to try and think about the specific model intervention that you could use for somebody is really helpful.”* - *“… So we set up a joint supervision group so that we could all bring cases… to make sure that the clinicians that are delivering this intervention feel well supervised, well supported and get the opportunity to learn from others.”* - *“Going to the peer supervision is really good because you're bouncing off other people who understand the models you're using and you've got ideas and understand the challenges you face and that can be really useful to just feel like you're not on your own in something and you're not trying to work it out on your own.”* - *“When you meet with others, and you talk with others about things, you get given a bit of ‘this is how you could implement this part’, or ‘this is how you could implement that part’. Or ‘if you finally want to cross from that bit to that bit, this is how I’ve managed to do that’. Those kinds of things. Having more of that available I think would help.”* - *“So I think good supervision really helped with that. [team lead], giving me the confidence for that. [team lead], just give it a go. It'll be fine using her experience and stuff. So I think the supervision alongside the structured nature of the protocol for me was really good.”* |
| Theme 2: Team culture, buy-in, and prioritisation of decisions | Teams differed greatly in the approach they took to implementing CT-PTSD, and this was largely driven by team culture and attitudes towards the intervention. This effected the extent to which the team prioritised the intervention, and took steps to embed it into their current systems and treatment plans. Sometimes teams were guided in this by their service lead, but sometimes it was in contrast to the service lead’s efforts. This could also be affected by the team’s views about their involvement in research. | |
| *Subtheme 1: Stabilisation and the meaning of individual therapy* | Stabilisation is an initial phase of therapy focused on risk reduction and emotion regulation. The team’s approach to this differed between teams and impacted their ability to implement CT-PTSD, with those that actively created strategies to move past stabilisation and onto the full treatment more successful in implementation. In addition, team’s views around the meaning of individual psychotherapy were also important for implementation. | - *“Rather than, you know, having an official stabilisation phase and an official trauma focus phase that I think if there's more investment in just building, trusting relationships with adults and I think just, I mean, stabilisation is just a how an easy framework because it gives something quick that they notice. Oh, that might make my life feel better. But I think the really important part is actually to trust and the relationship. But the stabilisation is a really nice thing to focus on. So, the intensity within the relationship is kind of reduced and helps them kind of coming with a focus rather than you because you're a bit dangerous.”* - *“So sometimes it's just focusing on the stabilisation and then the kind of symptoms have subsided and they don't want to move on to the reliving part…”* - *“I think even if we think about that kind of stage one or that kind of stabilisation phase of trauma-focused CBT, I think there is a focus within that on regulation and kind of an individual regulation work as opposed to you know, a lot of the young people that we're looking to support are still developing their understanding of coregulation with an adult and with a lot of support around them. So, I think some of the stuff within tf-CBT relies on a foundation that some of our young people don’t have.”* |
| *Subtheme 2: Competing therapies* | Some teams were also offering EMDR (Eye-Movement Desensitisation and Reprocessing). Some teams were able to provide both treatment options, and could effectively make decisions (sometimes with young people) on which treatment would be most appropriate. Other teams found it difficult to support both trauma-focused interventions, with some examples of active discouragement of CT-PTSD by EMDR-trained professionals. | - *“I did a joint appointment with them and she talked about offering EMDR or trauma-led CBT and she explained both of them to the young person. It was quite nice for the young person to have that choice of which one felt would be most appropriate to them.”* - *“The young person was finding she’s quite concrete and I think having something more visual written down in a word format than having to think in her mind or visualise and the bilateral stimulation she was just finding really quite ‘weird’ she said. And unusual. And she liked to talk a lot. So, I thought we could try trauma-focused CBT and then be sitting together and documenting so she can see exactly what we’re working through.”* - *“I’ve often heard kind of the EMDR therapists kind of say they preferred- they worried that the CBT re-traumatised people, whereas you know I’m less sure of that…”* |
| **Individuals** |  |  |
| Theme 1: Complexity of young people and therapist perception of readiness for treatment | MHPs frequently referred to the complexity of young people as a barrier to CT-PTSD, as it meant they were unable to engage young people in the trauma work. Many interpreted this complexity, or a general lack of motivation from the young person, as meaning that they were not ‘ready’ for trauma-focused work. However, others acknowledged that these factors are characteristic of a PTSD diagnosis, so it would be important to persist with the treatment. MHPs were able to deliver CT-PTSD despite complexity or reluctance from the young person, and reported that this was often helpful for the young person. | - *“It's putting together a bit of a picture of what's going on for the child at the time and what feels like a priority that to them in their life and what fits with school and education and things like that.”* - *“I've delivered the tf-CBT a number of times and my experience of young people each session is that they feel relief after talking about these things and actually their stability increases… when things don't go well, it's more because there's something that's keeping it stuck rather than the therapy itself.”* - *“You are thinking about timing of when people are wanting therapeutic support and I don't think I've had any young people in the last year that have said specifically they want to work on trauma-focused CBT. But I've had young adults that said they do.”* - *“And I just think that's just such a big ask of them at that point in their lives, developmentally.”* - *“A lot of the time, in conversation with the client there is quite strong resistance on focusing on the trauma itself and work might take too long with the timelines we have”* - *“Many of them don't want to do it because they don't want to think about the past. They want to think about the future”* - *“He’s able to open up more, he’s actually started talking about things that have happened to him … it’s taken a very long time for me to get him to feel like that.”* - *“It's complex, he's looked after and I would have thought that would, yeah, this would have been great. However, his presentation is such a mixed bag that it's really difficult to engage, but he is engaging. But I just think that because there's so many other aspects, I wouldn't really know where to start with that work with him because it would be focusing primarily on trauma.”* |
| Theme 2: Mental health professionals’ capability, confidence, and willingness | As mentioned in Theme 1, MHPs frequently commented on the complexity of young people coming through their service. They acknowledged that this resulted in some anxiety and hesitancy around CT-PTSD, and they often stated they did not know where to start, or did not know how to carry on with more difficult elements. MHP confidence improved after their first successful case, and was particularly helpful if this happened soon after the training to help consolidate learning. Some MHPs also acknowledged and addressed their avoidance, to help them deliver CT-PTSD. Some MHPs were not motivated to deliver CT-PTSD because it did not align with their own therapeutic approach. | - *“[..] That doesn't mean it can't work. It's just a constant dilemma that's in our minds and I'm trying to be really mindful to think about is it me that's being the resistance thing in terms of being too worried about the network and the safety and such like or is it actually just that as it is and it doesn't necessarily feel right or OK, but it is as it is and we have to.”* - *“I guess it could be confidence issues sometimes. If you're not familiar with using it, then that can be a concern. There is a lot to take in and I do frequently go back to the book and think and kind of like prepare two sessions ahead just in case it's someone who whizzes through or refuses to do something. And you know, I'm always in a situation where I think they're going to find out, that I don't actually know anything. I just read it in a book, you know. I guess I feel much more comfortable once we get to the trauma narrative”* - *“I don't think anybody feels that confident and kind of right- I've really got this.”* - *“And I think it's definitely been a confidence thing … After the training it's there, it's fresh isn't it? But without the repetition and without doing it you kind of lose it, don't you a little bit.”* - *“I'm not sure how, how confident I would feel going into a new piece of work. I think because so much time has passed since the training and where the first one didn't massively get kind of up and running it, it feels like that almost that that window where I would have been able to consolidate perhaps some of the training and go back and refresh myself. It feels like so much time has passed”* - *“…I think sometimes people can shy away from it because it is quite getting down to the nitty gritty of traumas that have happened so it can create a bit of anxiety for the clinician as well so it’s nice to have that support when we’re using the model”* - *“I hadn't done any trauma work like this kind of trauma-focused CBT in the past, done lots of CBT, but not trauma-focused, and my experience was I was very scared to do trauma work and I didn't want to make it worse and all those stereotypes and the training was fantastic and I think I have now been able to do 4 completed cases and then I've got another batch coming down the pipeline after my annual leave, which I wouldn't have felt the confidence and skill to do without the ADAPT trial. So from that perspective I thought it's been really, really good. I'm really grateful for that.”* - *“It is very oppositional to my approach, in that, as an Art psychotherapist, the emotional safety that I am creating with my young people is actually not talking about the trauma necessarily, it’s about talking about how that trauma can be shared through not talking. And then maybe some verbalisation may happen and I think that it’s not like we’re avoidant of the verbalisation it’s that it doesn’t have to be – it’s almost like secondary…”* |

*Note.* CLA refers to a child looked-after by the local authority (i.e., a young person in care); MHP = mental health professional (i.e., the participants in this study). CT-PTSD = Cognitive therapy for PTSD (i.e., the treatment focused on in this study).
